# Characterization and Quantification of Arsenic Species in Foodstuffs of Plant Origin by HPLC/ICP-MS

**DOI:** 10.3390/life13020511

**Published:** 2023-02-12

**Authors:** Teresa D’Amore, Oto Miedico, Ciro Pompa, Chiara Preite, Marco Iammarino, Valeria Nardelli

**Affiliations:** Department of Chemistry, Istituto Zooprofilattico Sperimentale della Puglia e della Basilicata, Via Manfredonia 20, 71121 Foggia, Italy

**Keywords:** arsenic, HPLC/ICP-MS, speciation, plant-based foods, infant foods, hazard identification

## Abstract

Arsenic is a well-known carcinogenic, mutagenic and toxic element and occurs in the environment both as inorganic arsenic (iAs) and organoarsenical compounds (oAsCs). Since the toxicity of arsenic compounds depends on their chemical form, the identification and determination of arsenic species are essential. Recently, the European Food Safety Authority, following the European Commission request, published a report on chronic dietary exposure to iAs and recommended the development and validation of analytical methods with adequate sensitivity and refined extraction procedures for this determination. Moreover, the authority called upon new arsenic speciation data for complex food matrices such as seaweeds, grains and grain-based products. Looking at this context, an optimized, sensitive and fast analytical method using high performance liquid chromatography followed by inductively coupled plasma—mass spectrometry (HPLC/ICP-MS) was developed for the determination of iAs (sum of arsenite—As^III^ and arsenate—As^V^) and the most relevant oAsCs, arsenobetaine, dimethylarsinic acid and monomethylarsonic acid. The method was validated with satisfactory results in terms of linearity, sensitivity, selectivity, precision, recovery, uncertainty, ruggedness and matrix effect, and then successfully applied for the analysis of several matrices, i.e., processed and unprocessed cereal and cereal products, fruits, vegetables, legumes, seaweeds, nuts and seeds. The results obtained indicate that not only seaweed and rice matrices but also many cereals, legumes and plant-based foods for infants and young children contain significant concentrations of iAs and oAsCs. These findings contribute to the data collection necessary to assess the role of these matrices in the total arsenic exposure and if specific maximum limits have to be established.

## 1. Introduction

Arsenic is a well-known carcinogenic, mutagenic and toxic element and occurs in the environment in four oxidation states, i.e., −3, 0, +3, and +5. Arsenite, As^III^, and arsenate, As^V^, are the predominant oxidation states, both as inorganic arsenic (iAs) and organoarsenical compounds (oAsCs). Monomethylarsonic acid (MMA), dimethylarsinic acid (DMA) and arsenobetaine (AB) are the most common organoarsenical compounds. In the terrestrial and marine ecosystem, iAs predominates in water and sediments, while a large number of oAsCs have been identified in the biotic community [1,2]. Apart from chemical forms and occurrence, these compounds greatly differ in their toxicity; particularly, iAs species are more harmful than oAsCs, with LD_50_ values from 100 to 500 times higher for the latter [3]. Drinking water and food products contribute most to the total exposure to arsenic for the general population, posing a severe health hazard [4,5,6,7].

For this reason, arsenic and iAs compounds were classified as “carcinogenic to humans”—Group 1 by the International Agency for Research on Cancer (IARC), while MMA and DMA were included in Group 2B—“possibly carcinogenic to humans”, based on the available toxicological evidence. AB, which is not metabolized in humans as well as other oAsCs, such as arsenolipids and arsenosugars, were defined as “not classifiable as to their carcinogenicity to humans”—Group 3 [1,8]. Although the endpoint of high concern is the carcinogenicity (skin, lung, bladder, liver, kidney and prostate cancers), several other harmful effects, observed in chronic toxicological studies, were described for arsenic compounds, including cardiovascular diseases, developmental toxicity, neurotoxicity, and even abnormal glucose metabolism and type II diabetes [9]. In this framework, in 2015, 2018 and 2022, the European Commission—EC released several Recommendations and invited the Member States to monitor the presence of iAs in a broad variety of foodstuffs and feed [10,11,12]. Similarly, in its recent report on chronic dietary exposure to iAs, the European Food Safety Authority—EFSA recommended the development and validation of analytical methods with adequate sensitivity and refined extraction procedures for this determination. The authority called upon new arsenic speciation data for complex food matrices such as seaweed products, foods intended for infants and young children, foods for special medical purposes, supplements and grains and grain-based products [13]. The same concern was previously reported by the World Health Organization—WHO. They expressed a pressing need for validated methods for selective extraction and determination of inorganic arsenic in food matrices and improved data on occurrence of different species of arsenic in, and their bioavailability from, different foods consumed [9]. These data are essential to enable an accurate estimation of exposure and to assess whether the contribution from these foodstuffs to the total exposure of arsenic would require the establishment of specific maximum limits (MLs). In fact, the current European legislation, Regulation (EC) No. 1881/2006, sets the MLs for iAs (sum of As^III^ and As^V^) only in rice and rice-based food items, included those intended for infants and young children [14,15].

The main techniques described for the analysis of arsenic species are based on the combination of separation techniques such as liquid chromatography (LC), gas chromatography (GC) or capillary electrophoresis (CE) and element-selective detectors (e.g., ICP-MS, ICP-OES, AAS, ESI-MS, TOF-MS, AFS); currently, ICP-MS is the most used technique due to the high sensitivity, wide linear range and low detection limit [3,16,17,18,19,20]. In addition, high standardized extraction procedures are needed, since sample preparation may be the “bottleneck” in the entire protocol of arsenic species determination [21,22].

The aim of the present study was to develop an optimized and sensitive analytical method allowing fast and efficient identification and quantitation of iAs and the relevant oAsCs by high performance liquid chromatography followed by inductively coupled plasma—mass spectrometry (HPLC/ICP-MS) detection. The extraction recovery experiments were performed by comparing two different extraction devices (shaking water bath and ultrasonic bath). A complete validation study in terms of linearity, sensitivity, selectivity, precision, recovery, matrix effect and matrix ruggedness was carried out. A comparison of the main characteristics of the developed method with some established methods for speciation and determination of arsenic was presented. The developed method was successfully applied for the analysis of several plant-based matrices, selected reflecting not only the EC and EFSA requests, but giving a special emphasis to novel consumption habits and food choices of certain consumers and subsets of populations (e.g., foods for special nutrition, food supplements, high protein, high nutrient plant matrices for vegans, vegetarians and flexitarians). Indeed, since the consumption of plant-based commodities is exponentially growing, the related exposure to arsenic can be relevant. Therefore novel, simplified and fast methods, which can be applied to a wide range of food matrices, should be implemented. In this context, this method may be very attractive for routine analysis of arsenic species, and also as a useful tool in occurrence and monitoring studies. These data are needed to fill uncertainties and data gaps, and to develop exposure assessment and risk characterization, and eventually to introduce specific risk management measures.

## 2. Materials and Methods

### 2.1. Reagents and Working Standard Solutions

High-purity reagents, trace element grade concentrated nitric acid (HNO_3_, 68% *v/v*) and hydrogen peroxide (H_2_O_2_ 30% *v/v*) were supplied by Romil Ltd., Cambridge, UK. Ultrapure Milli-Q^®^ water (H_2_O, 18.2 MΩ cm^−1^, at 25 °C) was used to prepare all reagents and standards. Sodium hydroxide solution (NaOH, 50% *w/v*), sulfuric acid (H_2_SO_4_, 20% *v/v*) and sodium chloride (NaCl, ACS reagent, ≥99.0%) were acquired from Merck KGaA (Darmstadt, Germany). Methanol (CH_3_OH) of LC-MS grade (Carlo Erba Reagents, Rodano, Italy), ammonium hydroxide (NH_4_OH, 30% *v/v*) and ammonium bicarbonate (NH_4_HCO_3_, ReagentPlus^®^, ≥99.0%) (Sigma-Aldrich Co. LLC, Steinheim, Germany) were used to prepare the mobile phase (50 mM NH_4_HCO_3_, 3% *v/v* CH_3_OH, pH 10.3, 25 °C).

Diarsenic trioxide, (As_2_O_3_, 197.84 g mol^−1^, ≥99%), sodium arsenate dibasic heptahydrate (Na_2_HAsO_4_·7H_2_O, 312.01 g mol^−1^, ≥98.0%), sodium dimethyl arsenate trihydrate (C_2_H_6_AsO_2_Na·3H_2_O, cacodylic acid, sodium salt, trihydrate, 214.03 g mol^−1^, ≥98%), sodium methyl arsenate hexahydrate (CH_3_AsO_3_Na_2_·6H_2_O, 291.90 g mol^−1^, ≥97.5%) and arsenobetaine (C_5_H_11_AsO_2_, 2-(trimethylarsonio)acetate, 178.06, g mol^−1^, ≥95.0%) were obtained from Sigma-Aldrich Co. LLC (Steinheim, Germany). Standard stock solutions of Na_2_HAsO_4_·7H_2_O, C_2_H_6_AsO_2_Na·3H_2_O, CH_3_AsO_3_Na_2_·6H_2_O and C_5_H_11_AsO_2_ at concentration of 1000 µg L^−1^ were obtained by weighing each analyte using analytical balance and dissolving the powder in H_2_O, with the addition of 0.5% NaOH *w*/*v* as a stabilizing agent. Standard stock solution of As_2_O_3_ at a concentration of 1000 µg L^−1^ was prepared by dissolving the powder in 20% NaOH *w*/*v* solution, then neutralized with 20% *v*/*v* H_2_SO_4_, and finally diluted to 1000 mL with 1% *v*/*v* H_2_SO_4_. The solutions were stored at 4 °C for a period of up to 6 months. Working standards at concentrations of 10.0, 2.0, 0.5, 0.1, 0.05 µg L^−1^ were freshly prepared before the analysis by appropriate dilution in 0.5% *w*/*v* NaOH solution. Ultrapure argon (Ar, 99.9999% purity) was obtained from SAPIO s.r.l. (Monza, Italy). As standard solution (1000 mg L^−1^) for instrumental tuning was provided by VWR international Ltd. (Leicestershire, England). The National Institute of Standards and Technology (NIST, Gaithersburg, MD-USA) supplied the Standard Reference Material—SRM 1568b, rice flour, used for the validation study and analytical quality controls.

### 2.2. HPLC/ICP-MS Analysis

Chromatographic separation was performed on a HPLC system, Flexar^™^ FX-15 equipped with binary chromatographic pump, thermostated autosampler, solvent manager and column oven (Perkin Elmer, Waltham, MA, USA). A PSDVB/Trimethylammonium anion exchange column (PRP-X100 Anion Exchange HPLC Column, PEEK, 2.1 × 250 mm, 5 µm, Hamilton, Boston, MA, USA) and alkaline elution conditions were chosen for ensuring the best separation of analytes in 7 min, after testing different isocratic and gradient elutions. One minute of washing with HNO_3_ 4% *v/v* was added in order to minimize the phenomena of matrix and carbonate deposition on the sample introduction system. A SympHony pH-meter, supplied from VWR International (West Chester, PA, USA), using a combined glass electrode was used for the pH measurement of the mobile phases. The analytes were detected by the inductively coupled plasma mass spectrometer (ICP–MS) PerkinElmer NexION^®^ 2000 (Waltham, MA, USA), equipped with a concentric nebulizer (Meinhard Associates, Golden, CO, USA), a baffled quartz cyclonic spray chamber (Glass Expansion, Inc., West Melbourne, Australia), a demountable quartz torch with a 2.0 mm internal diameter quartz injector tube and a quadrupole ion deflector (QID). The nebulizer gas (Ar) flow rate was set to 1.00 L min^−1^, plasma gas (Ar) flow rate to 15.0 L min^−1^, auxiliary gas flow rate to 1.0 L min^−1^ and radio frequency RF power to 1600 W. The QID voltage was prior tuned to the analyte mass by infusing a 1.0 ng mL^−1^ As solution in mobile phase. The chlorine channel (Cl-35) was monitored for potential polyatomic interferences (ArCl^+^ and CaCl^+^, *m/z* 75).

The chromatographic and spectrometric instrumental set-up is summarized in Table 1.

### 2.3. Sample Preparation

A representative sample portion of 50–100 g, depending on the sample availability, was powdered in a mixer for 1 min at room temperature. A test portion of 0.200 to 0.500 ± 0.005 g powdered sample was weighed using an analytical balance (Mettler Toledo s.p.a., Milan, Italy) into a 15 mL centrifuge tube and 10.0 mL of extraction solution (HNO_3_ 0.1 N in 3% *v/v* H_2_O_2_) were added. The sample was suspended in the extractant by vortexing for at least 1 min at 1500 ***g*** and placed in shaking water bath, heated at 90 °C for 2 h. In these conditions, all iAs species were oxidized to As^V^. After cooling, the sample was centrifuged at 4500 *g* (10 min, 10 °C) and the collected supernatant was transferred into a 15 mL polypropylene tube. Two mL of the supernatant were filtered through 0.45 μm Minisart^®^ NML cellulose acetate syringe filter (Sartorius, Goettingen, Germany) into a HPLC polypropylene vial. The extraction and the analysis of each sample were performed twice, and the concentrations obtained were reported as the mean of the two replicates.

### 2.4. Validation Study

The method validation is an integral part of good analytical practice. It is also an essential and general requirement of the European rules for the official control methods, ISO 17025:2017 and Regulation (EU) No. 625/2017, to determine an analytical procedure as suitable or else “fit for purposes” [23,24]. A single laboratory study, in-house validation model, in agreement with Commission Decision No. 2002/657/EC was used for the determination of performance characteristics of the optimized method [14,25,26]. The parameters evaluated for analytical method validation were linearity, selectivity, limit of detection (LoD) and limit of quantification (LoQ), accuracy, matrix ruggedness, matrix effect and measurement uncertainty. The assessment of accuracy was performed following ISO 5725–2, as a sum of within-lab reproducibility (or intermediate precision) and trueness [27]. Both a rice flour standard reference material, SRM NIST-1568b, and spiked samples were used for a complete accuracy assurance. All samples were spiked prior to extraction. In Table 2, the measurement method for the determination of validation parameters is described.

**Table 2:** Validation study.

Performance Characteristics	Evaluation/Measurement Approach
Linearity Working Range	Injection of five iAs and oAsCs standard solutions in extractant 0.05, 0.1, 0.5, 2.0, 10.0 μg L^−1^ Three replicates at each concentration level, injected in three different analytical sessions, with the same instrument, performed on different days and operators); n = 3 Regression of calibration curve with the least square method Mandel’s fitting test to check linearity Calculation of determination coefficient value, acceptable if R^2^ ≥ 0.99
Selectivity	Analysis of 15 pseudo-blank samples, in two replicates under repeatability conditions
Limit of detection Limit of quantification	Estimation of LoD via calibration approach: injection of iAs and oAsCs standard solutions in extractant 0.05, 0.1, 0.5, 2.0, 10.0 μg L^−1^ (two replicates at each concentration level) Construction of mean calibration curve and usage of calibration function to estimate the standard deviation of intercept and the slope (1) LoQ=(10×σib) (2) LoD=(3.3×σib) where: *σi* is the standard deviation of intercept *b* is the slope of the calibration function
Precision and trueness	Analysis of a blank rice sample fortified at two levels: 15.0 and 30.0 μg kg^−1^ with a mix of iAs and oAsCs standard solution (6 replicates in 2 different working sessions with the same instrument, different days, operators and instrumental calibrations) Evaluation of relative standard deviation for each analyte and recovery values Usage of a SRM NIST-1568b for the assessment of trueness: recovery values obtained on samples spiked at 15.0 and 30.0 μg kg^−1^ were used to correct the results of 6 independent tests; n = 18
Measurement uncertainty	Maximum standard uncertainty approach: (3) Uf=(LoD2)2+(α×C)2 where: *U_f_* is the maximum standard uncertainty (μg kg^−1^) α = numeric factor depending on the value of C
Matrix effect	Calibration graph method: the ratio between the slope of the curve obtained for the matrix-matched extracts and the slope of the curve for the standard calibration curve minus 1, expressed in percentage; n = 3 (4) ME=(Slopematrix Slopesolvent−1)×100
Matrix Ruggedness	Change of matrix to analyse: conditions of *major changes;* 10 pseudo-blanks and 6 additional experiments for 3 different pools of samples of legume, cereal and vegetable powders at 30.0 μg kg^−1^ in matrix. Comparison of precision and recovery data with the results obtained for validation matrix

### 2.5. Interlaboratory Comparison: Proficiency Test Round

The reliability and accuracy of the developed HPLC/ICP-MS method were further evaluated by an external quality assessment, i.e., proficiency test—PT, as prescribed by the Regulation UNI CEI EN ISO/IEC 17025:2017 [23]. The PT materials were supplied by Fapas^®^ (Fera Science Ltd., York, UK), a food chemistry PT provider, accredited in agreement with the general prerequisites of ISO/IEC 17043:2010, and consisted of (1) powdered brown rice and (2) infant cereal, both naturally contaminated [28]. The samples were analyzed for the quantification of iAs and other trace/oligo elements (tAs, Cd, Cr, Fe, Hg, Ni, Pb, Se, Zn) by a previous validated method [29]. Participants were (1) from 41 to 106 and (2) from 21 to 53 for each analyte.

The analysis was carried out in duplicate, and the results were calculated as the mean of two measurements. For the evaluation of standard score, the Z-test, satisfactory if |z| ≤ 2, was used.

z = (x − x_a_)/σ_P_ (5)

where x is the participant’s reported result, x_a_ is the assigned value and σ_P_ is the standard deviation for proficiency.

### 2.6. Software and Statistical Analysis

Empower 3 (Waters, Milford, MA, USA) software was used for acquisition, processing identification and quantification of data, while ICP-MS was controlled by Syngistix^TM^ 2.5 (Perkin Elmer, Waltham, MA, USA). Statistical analysis was used for the assessment of method linearity, as reported in Table 2. The one-way analysis of variance (ANOVA, *p* < 0.05) was used for comparing the data at each fortification level in terms of recovery percentage and relative standard deviation (RSD%). This comparison is needed for checking out the homoscedasticity of values obtained at different levels.

For descriptive analysis, the upper bound substitution approach was used for treating left-censored data [30].

## 3. Results and Discussion

### 3.1. Procedure Optimization

**Figure 1:**
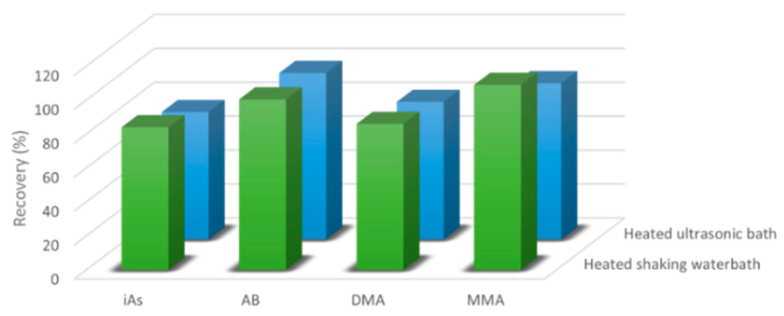
Extraction optimization: comparison of two techniques: heated shaking water bath at 90 °C for 2 hrs and heated ultrasonic bath at 80 °C for 2 h.

The laboratory started from the European standard method EN-16802, which described a method for determination of iAs, then developed an optimized analytical procedure [31]. In fact, generally for food safety applications, the speciation of As is limited to total iAs, due to similar toxicity of As^III^ and As^V^ and the instability of As^III^, which evolves into the oxidized form during food storage and preparation. Several extraction methods were described in the literature, i.e., microwave assisted extraction, shaking and sonication using different mixtures of solvents [16,17,18,19,20,21]. In order to fulfil the legal requirements, the laboratory selected an acid and oxidant solution (HNO_3_ 0.1 N in 3% *v*/*v* H_2_O_2_) for extraction of both iAs and oAsCs. Two different extraction volumes (10 and 20 mL) and techniques (heated shaking water bath at 90 °C for 2 hrs and heated ultrasonic bath at 80 °C for 2 h) were tested. Six samples (two rice, two infant foods, two seaweeds) and two procedural blanks were fortified (30.0 μg kg^−1^) with (1) As^III^ only, (2) DMA only, (3) AB only, (4) MMA only, (5) As^V^, (6) mix of six species. The complete oxidation of As^III^ to As^V^ was achieved with both techniques. In these conditions, the remaining oAsC species were not degraded or interconverted in others. Although similar recovery values were achieved for both techniques, the best compromise in terms of extraction efficiency, solvent consumption and sample weight for all analytes was obtained with heated shaking water bath extraction at 10 mL, 90 °C for 2 h, as shown in Figure 1. The data obtained from recovery experiments were further checked out using the SRM NIST-1568b, as described in Table 2.

**Figure 2:**
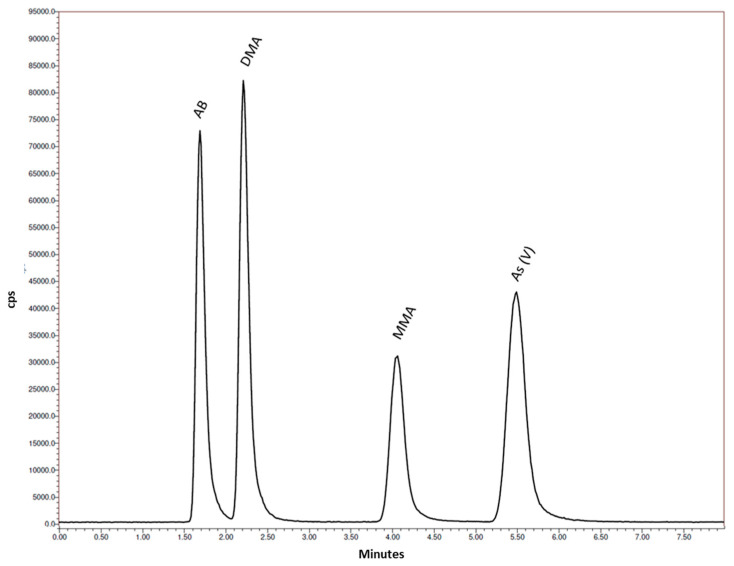
Chromatograms of standard solution (2.0 µg L^−1^).

The technique most described in literature for chromatographic separation is liquid chromatography easily coupled with several detectors (e.g., HG-AAS, HG-AFS, ICP-OES, and ICP-MS, ESI-MS), using anionic or, less frequently, cationic conditions. In this study, anionic conditions, including a strong anion exchange column with quaternary ammonium [-N^+^(CH_3_)_3_] as functional group and alkaline elution (50 mM NH_4_HCO_3_ in 3% *v*/*v* CH_3_OH), were chosen, exploiting the low pka values of the arsenic species under investigation [3,22,32]. Three different mobile phase pH were evaluated (9.5; 10.0; 10.3) and the best conditions of separation of analytes, retention time repeatability (RSD% < 1.2; n = 10) and peak shape were observed using the last pH value. The usage of different percentages of carbon donor solvents was often described in literature especially for arsenic and selenium speciation, since they may enhance signal intensities due to the well-known “carbon-induced signal enhancement” phenomenon. However, several authors reported a possible overestimation of As concentrations due to an excessive amount of CH_3_OH mobile phase. Similarly, both carbon solvents and matrices may induce instrumental drift which is considered one of the main problems of As speciation repeatability during the time [33,34]. For this reason, a minimal percentage of CH_3_OH (3% *v*/*v*) was used in the mobile phase, further diluted with 15% mobile phase B (ultrapure water). Furthermore, after the 7 min necessary to obtain the complete separation of analytes, 1 min more of washing with HNO_3_ 4% *v*/*v* was added, adjusting the switching valve settings, to wash the sample introduction system (nebulizer, cyclotronic chamber, torch). For the detection and quantification of As species, undoubtedly ICP-MS is the most efficient technique, permitting the achievement of high selectivity and low LoD and LoQ. Moreover, the monitoring of As-75, instead of other species (e.g., ^75^As^16^O^+^), permits reaching a better sensitivity [32,35]. These characteristics make this method particularly useful both for routine analysis and for specific monitoring studies (e.g., total diet studies), where low limits of detection allow minimization of the percentage of undesired left-censored data. In Figure 2, a standard solution chromatogram is shown.

### 3.2. Method Validation

The analytical performances of the developed method were evaluated in terms of linearity, selectivity, LoD and LoQ, accuracy (precision and trueness), matrix effect, ruggedness and measurement uncertainty. The assessed parameters were in agreement with the European guidelines and requirements assumed as reference in this study [25,26,36]. The evaluation of method linearity was carried out by the Mandel’s fitting test. The determination coefficients, calculated from the calibration curves, were higher than 0.99 for all the analytes considering both the mean and the single curves. The LoQ values were 0.075 for iAs, 0.241 for MMA, 0.235 for DMA, 0.321 µg kg^−1^ for AB. The selectivity of the method was verified by analyzing 15 pseudo-blank samples, i.e., native test sample in which the analyte is present at a concentration level close to (but not exceeding five times) the expected LoD, as suggested by the European Union Reference Laboratories (EURL) for Heavy Metals in Feed and Food (EURL HM), for Polycyclic Aromatic Hydrocarbons (EURL PAH), for Mycotoxins (EURL Mycotoxins), and for Dioxins and PCBs in their Technical Report on the estimation of LoD and LoQ for measurements in the field of contaminants in feed and food [37]. The Kolmogorov–Smirnov test was used to process the data obtained from precision and recovery experiments, in order to check the distribution normality. The intermediate precision was expressed as RSD% and was <7.35% for all analytes. The recoveries were in the range 80–120% and they were used as correction factors in the analyses of samples. The calibration graph method was used for calculation of the matrix effect (ME), expressed in percentage, for each analyte. A value of 0% indicates no ME, while values of <0% and >0% indicate ionization suppression and enhancement, respectively. Generally, no ME correction factor was applied if it was ≤|25%|. In this study the ME was between 9 and 19%, so the standard calibration curve in solvent was used for the analysis of commercial samples. In Table 3, the validation parameters are reported.

### 3.3. Interlaboratory Comparison: Proficiency Test Round

The laboratory analyzed the two matrices provided, i.e., powdered brown rice and infant cereal, both declared as naturally contaminated by the provider, in July and October 2021, respectively. The PT results are presented in Table 4. Looking at the available optional procedure details, the laboratory was among participants using minimal sample size and solvent volume for extraction, in agreement with principles of green analytical chemistry [38]. Most part of participants used HPLC/ICP-MS for the analysis of iAs, while only few laboratories used other techniques (e.g., liquid chromatography—atomic fluorescence spectrometry, hydride generation—ICP—optical emission spectroscopy). The satisfactory z-score values for both matrices, particularly for iAs (0.1 for and 0.3 for), confirmed the reliability and the accuracy of the developed method for the determination of iAs in foods.

### 3.4. Comparison with Other Methods

Over the years, several approaches for arsenic speciation analysis were developed and various modifications were advanced. An overview of some recent and innovative procedures is summarized in Table 5. Different extraction protocols, as well as comparison studies of their efficiency, were extensively described in the literature. Microwave assisted extraction or digestion (MAE and MAD, respectively), were used in several studies since they ensure high recovery [17,35]. However, they often require large amounts of acid solvents. Ma et al. compared three common extraction methods (shaking, sonication and microwave) for the extraction of arsenic species in leafy vegetables, obtaining high efficiency with MAE [21]. Although most parts of recently developed methods are based on LC or ion chromatography (IC) coupled with mass spectrometers as the analyzer, due to its sensitivity and precise quantitation (rarely with optical emission spectrometry, due to it less sensitivity), other analytical approaches are also described. Yang et al. developed a sheath–flow interface to couple CE with ICP-MS to characterize arsenic species from seafood [39]. On the other hand, some protocols based on GC and tandem mass spectrometry were developed for the determination of inorganic arsenic species (As^III^ and As^V^), previous derivatized using dimercaprol, in rice products and rice based infant foods, reaching very low LoDs [40,41]. Both Guillod-Magnin et al. and Lin et al. developed an IC-ICP-MS method but using an anion exchange column and cation exchange column, respectively, and obtained a perfectly inverted chromatographic profile. In particular, in the second study, two unidentified peaks were found in some shellfish samples [42,43]. Other methods used hydride generation coupled with ICP—triple quadrupole bypassed the chromatographic separation with very good results in terms of recoveries [44]. A very interesting procedure was developed by coupling laser ablation with the ICP-MS (LA-ICP-MS) for direct measurement in solid samples of inorganic arsenic species previously separated by thin layer chromatography [45].

Most parts of these procedures were highly optimized only for a few matrices. In addition, the main validation parameters of the developed method were also compared with these established methods for speciation and determination of arsenic species. In this validation study, in particular, not only “classical” parameters were evaluated but also matrix effect and ruggedness. With these investigations, the applicability field of the novel method was extended to all matrices requested both by the EU Commission recommendation and EFSA/WHO reports. Indeed, the main goal of this analytical procedure was the improvement of reliability, the standardization and the simplification of processes in order to ensure the accuracy, robustness and homogeneity of data.

### 3.5. Application to Commercial Samples

The proposed analytical method was employed to investigate iAs and oAsCs contamination in 42 commercial plant-based samples (9 rice and rice products, 6 cereals, 5 vegetable powders, 2 legumes, 12 cereal-based food for infants and young children, 7 seaweeds and seaweed supplements, and 1 rice supplement). The samples were analyzed in duplicate, and the concentration was calculated as the mean of two measurements. In Table S1, the concentrations of iAs, MMA, DMA and AB, and the details about ingredients and the origin of products are reported. Mean concentrations and standard deviation for each food category are also shown. Generally, iAs and DMA are the most representative species, quantifiable in 100% and 86% of samples, with a mean content of 173 and 61 µg kg^−1^, respectively. AB was detected only in two seaweeds, confirming its prevalence in the marine environment. Considering the European regulatory framework, one sample of cereal-based food for infants and young children was above the MLs with an iAs content of 125 µg kg^−1^ [14,15]. It was a rice cream made from 94% rice flour with the addition of various micronutrients, vitamins and minerals, as laid down by the nutritional requirements described in the European Commission Directive No. 2006/125/EC [46].

**Figure 3:**
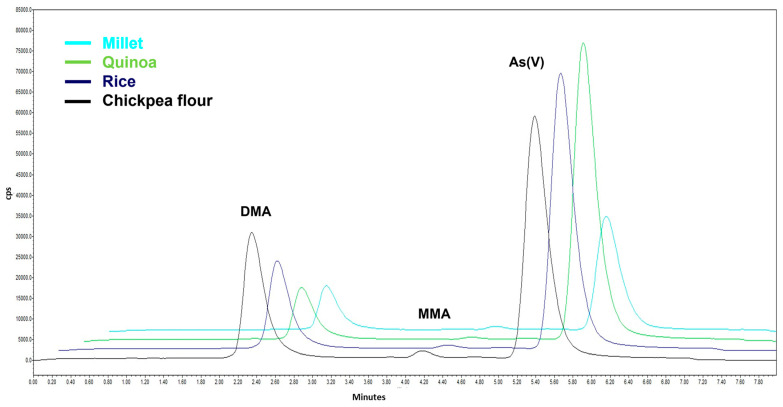
Chromatogram comparison of commercial samples: (teal) millet (DMA: 7.37, MMA: 1.05, iAs: 163.87 µg kg^−1^), (green) quinoa (DMA: 12.0, iAs: 217.8 µg kg^−1^), (blue) rice (DMA: 18.0, MMA: 0.7, iAs: 222.9 µg kg^−1^), (black) chickpea flour (dilution 1:2, DMA: 33.3, MMA: 0.9, iAs: 462.6 µg kg^−1^).

Cereal and rice categories had a similar mean concentration of iAs, 87 µg kg^−1^ and 105 µg kg^−1^, respectively, whereas rice-based commodities showed a DMA concentration four times higher than cereals (19 and 5 µg kg^−1^, respectively), similar to the occurrence data provided by the EFSA report [13]. It is noteworthy that rice and rice-based products show a parallel increasing trend of the iAs and rice content. In fact, the results vary from 9 µg kg^−1^ of an infant food sample containing 17% of rice to 223 µg kg^−1^ in wholegrain red rice containing 100% of rice. In spite of the restricted number of analyzed samples, the results are comparable with other studies (dietary exposure, total diet and monitoring) carried out worldwide [4,18,42,47,48]. However, other grains (millet, quinoa and oat) seem to accumulate more iAs than others (corn). This trend is also confirmed for infant foods. Vegetable powders are characterized by a very low content of iAs, except for a sample of moringa leaves (iAs: 60; DMA: 27 µg kg^−1^). In a chickpea flour sample from organic agriculture, the concentration of iAs was high (463 µg kg^−1^), underlining the necessity of more investigations and specific monitoring studies for these matrices. Indeed, according to FAO statistics, their demand and consumption are globally increasing, as well as their emerging applications in plant-based meat alternatives to address the protein needs of vegetarian/vegan/flexitarian consumers or also people who reduce meat due to health/environmental reasons [29,49,50,51,52]. Taking into account the product origin, no significant differences in iAs and oAsCs levels were observed between UE and extra-EU products. The chromatograms of the most interesting samples are shown in Figure 3.

A distinct discussion is needed for seaweeds. In fact, the concentration of As species in seaweeds was high and oAsCs were differently distributed, due to morphological variability and structural complexity of these matrices. In general, the concentrations found (range: iAs: 2–2718; DMA: 0.4–1118; MMA: 0.2–153: AB: 0.3–99 µg kg^−1^) were in agreement with previous studies and confirmed that taxonomy plays a significant role in the content of iAs and oAsCs [32,53,54]. In particular, the two samples of brown seaweeds (*Fucus vesiculosus*, *Ecklonia bicyclis*) had a higher content of iAs than green (*Chlorella pyrenoidosa*) or red seaweeds (*Chondrus crispus*, *Palmaria palmata*) (Table S1). Moreover, several studies indicated that the content of other organic forms, i.e., arsenosugars and arsenolipids, may be relevant in these matrices. However, the determination of these compounds was not performed due to the unavailability of commercial standards [19]. According to FAO and WHO, the world production of seaweeds has more than tripled since the turn of the millennium, and similarly their consumption (direct ingestion or as food supplements) and usage in livestock and aquaculture feed supplementation are exponentially growing. Thus, the implementation of the regulatory framework and the establishment of MLs for iAs and oAsCs seems particularly urgent, especially for seaweed products [11,55].

## 4. Conclusions

In this work, a fast, optimized and sensitive analytical method for the determination of iAs, AB, DMA, MMA in foodstuffs of plant origin by high performance liquid chromatography followed by inductively coupled plasma—mass spectrometry was developed, refined and validated. Their extraction was successfully implemented by means of a heated shaking water bath. The ammonium bicarbonate-based elution was optimized to assure the best separation of the analytes in 7 min with high selectivity. The method was fully validated in terms of linearity (R^2^ ≥ 0.99), LoD (0.025–0.106 µg kg^−1^) and LoQ (0.075–0.321 µg kg^−1^), selectivity, precision (RSD ≤ 7.3%), recovery (81–118%) and measurement uncertainty (18.2–22.0%). An in-depth investigation of the matrix effect and matrix ruggedness ensured high reliability, applicability and efficiency. The optimized method was applied for the analysis of 42 commercial samples. A preliminary monitoring study was also presented. Through these characteristics, this study will contribute to the fast and accurate determination of the most relevant arsenic species in plant-based matrices, and to the collection of data necessary to assess the role of these matrices in the total arsenic exposure.

## Figures and Tables

**Table 1:** Instrumental set-up.

Parameter	HPLC Conditions
Column	UPLC PRP-X100 Anion Exchange HPLC Column—i.d. 2.1 mm, l. 250 mm, p.s. 5 µm
Mobile phase	Isocratic elution A/B (85:15) A: 50 mM NH_4_HCO_3_ in CH_3_OH 3% *v/v* B: ultrapure water
pH	10.3
Flow rate	0.35 mL min^−1^
Run time	7 min + 1 min washing
Column T	25 °C
Autosampler T	20 °C
Diverter valve	0–7 min from HPLC to ICP; 7–8 min form HPLC to waste
Injection volume	30 µL
	**ICP-MS Conditions**
RF power	1600 W
Sample introduction system	Meinhard concentric PTFE nebulizer High Purity Quartz Cyclonic Spray Chamber
Plasma gas flow	15.0 L min^−1^
Aux gas flow	1.0 L min^−1^
Peristaltic pump control	sample flush 60 s; sample flush speeding −35 rpm
Isotopes monitored	As-75; Cl-35
Dwell time	As: 450 ms; Cl: 50 ms
Mode	Standard
Quadrupole Ion Deflector	Off

**Table 3:** Validation parameters.

Parameter	iAs	AB	DMA	MMA
Linearity R^2^	≥0.99	≥0.99	≥0.99	≥0.99
Range µg kg^−1^	0.025–400	0.106–400	0.079–400	0.077–400
LoQ µg kg^−1^	0.075	0.321	0.241	0.235
LoD µg kg^−1^	0.025	0.106	0.079	0.077
Precision (mean) RSD% *	4.96	7.35	3.15	4.92
Recovery (mean) R% *	81.3	100.4	85.9	117.8
Uncertainty U%	18.2–22.0	18.2–22.0	18.2–22.0	18.2–22.0
Selectivity	Verified for plant-based processed and unprocessed foods (cereals, fruits, vegetables, tubers, legumes, seaweeds, nuts and seeds)			
Matrix Effect ME%	<9%	<16%	<12%	<19%
Matrix Ruggedness	Verified for plant-based processed and unprocessed foods (cereals, fruits, vegetables, tubers, legumes, seaweeds, nuts and seeds)			

RSD: relative standard deviation; LoQ: limit of quantification; LoD: limit of detection; * n = 18.

**Table 4:** Proficiency test results.

Matrix	Analyte	Result µg kg^−1^	Assigned Value µg kg^−1^	z Score	σ_P_
Powdered Brown Rice (1)	Arsenic (total)	687.0	643.2	0.4	110
	Arsenic (inorganic)	119.0	117.5	0.1	25.9
	Cadmium	27.8	25.3	0.4	5.58
	Iron	10700	10100	0.5	1.14
	Lead	51.2	48.3	0.3	10.6
	Nickel	586.0	479.5	1.2	85.7
	Zinc	13600	14600	-0.4	1.53
Infant Cereal (2)	Arsenic (total)	128.0	113.0	0.6	24.9
	Arsenic (inorganic)	94.0	88.5	0.3	19.5
	Cadmium	35.3	32.8	0.3	7.23
	Chromium	149.0	126.7	0.8	27.7
	Lead	49.1	44.9	0.4	9.89
	Mercury (total)	29.6	33.1	0.5	6.51
	Selenium	67.5	78.3	-0.6	17.2

σ_P_: standard deviation for proficiency.

**Table 5:** Recent instrumental methods for the determination of arsenic species.

References	Extraction	Detection	Analytes	Matrices	Recovery (%)	LoD	Validation Parameters	Notes
Vu et al. (2019) [35]	MAD	HPLC-ICP-DRC-QMS	AB, DMA, MMA, As^III^, As^V^	rice	70.0–135.5	0.5–2.9 ng g^−1^	linearity, recovery, LoD, LoQ	species monitored ^75^As^16^O^+^
Ma et al. (2017) [21]	Shaking UAE MAE	HPLC-ICP-MS	AB, AC, DMA, MMA, As^III^, As^V^	leafy vegetables	-	-	extraction efficiency (%), LoD, LoQ	different extraction protocols
Jeong et al. (2017) [17]	-	HPLC-ICP-MS	DMA, MMA, As^III^, As^V^, DMDTA, DMMTA	water	85.1	0.04–0.26 µg L^−1^	linearity, recovery, LoD, LoQ	reversed phase C18 column; confirmation of DMDTA and DMMTA by ESI-MS
Guillod-Magnin et al. (2017) [42]	oven-heated SLE	IC-ICP-MS	DMA, MMA, As^III^, As^V^	rice and rice products	100–117	0.29–2.45 µg kg^−1^	linearity, recovery, LoD, LoQ, trueness, precision	
Lin et al. (2020) [43]	MAE	IC-ICP-MS	AB, DMA, MMA, As^III^, As^V^	seafoods, seaweeds	92–103	0.08–0.12 ng g^−1^	linearity, LoD, LoQ trueness, precision	cation exchange column
Kisomi et al. (2020) [45]	-	µTLC-LA-ICP-MS	As^III^ and As^V^	water	71–101	0.037–0.27 µg kg^−1^	linearity, trueness, precision, matrix effect	
Jung et al. (2018)/Jung (2017) [40,41]	water bath	GC-MS-MS	As^III^ and As^V^	ready-to-eat rice products, rice based infant foods	90–117	0.0159 ng g−1	linearity, selectivity LoD, LoQ trueness, precision	derivatization reagent: BAL
Yang et al. (2009) [39]	MAE (CH_3_OH–H_2_O 1:1 *v/v*)	CE-ICP-MS	DMA, MMA, As^III^, As^V^	*Mya arenaria* Linnaeus; shrimps	96–105	1.0–1.9 µg kg^−1^	linearity, precision, recovery	sheath–flow interface to couple CE with ICP-MS
Musil et al. (2014) [44]	MAD	HG-ICP-QQQ	DMA, As^III^, As^V^	rice, seafoods, seaweeds	95.8–100.6	0.9–1.1 µg kg^−1^	linearity, precision, recovery	derivatization reagent: NaBH_4_ and HCl
This method	shaking water bath UAE	HPLC-ICP-MS	AB, DMA, MMA, sum of As^III^ and As^V^	cereals, fruits, vegetables, tubers, legumes, seaweeds, nuts, seeds, supplements, infant foods	81.3–117.8	0.025–0.106 ng g^−1^	selectivity, linearity, LoD, LoQ, trueness, precision, matrix effect, measurement uncertainty, ruggedness	

MAD: microwave assisted digestion; MAE: microwave assisted extraction; UAE: ultrasonic assisted extraction; SLE: solid–liquid extraction; HPLC-ICP-DRC-QMS: high performance liquid chromatography—inductively coupled plasma dynamic reaction cell quadrupole mass spectrometry; HPLC-ICP-MS: high performance liquid chromatography—inductively coupled plasma mass spectrometry; IC-ICP-MS: ion chromatography—inductively coupled plasma mass spectrometry; µTLC-LA-ICP-MS: µThin layer Chromatography—Laser Ablation Inductively coupled Plasma Mass Spectrometry; CE-ICP-MS: capillary electrophoresis—inductively coupled plasma mass spectrometry; GC-MS-MS: gas chromatography- tandem mass spectrometry; HG-ICP-QQQ: hydride generation—inductively coupled plasma mass spectrometry—triple quadrupole; ESI-MS: electrospray ionization-mass spectrometry; AB: arsenobetaine; AC: arsenocholine; MMA: monomethylarsonic acid; DMA: dimethylarsinic acid; As^III^: arsenite; As^V^: arsenate: DMDTA: dimethyldithioarsinic acid; DMMTA: dimethylmonothioarsinic acid; LoD: limit of detection; LoQ: limit of quantification; BAL: British Anti-Lewisite.

## Data Availability

The authors confirm that the data supporting the findings of this study are available within the article and its supplementary materials.

## Supplementary Materials

The following supporting information can be downloaded at https://www.mdpi.com/article/10.3390/life13020511/s1, Table S1: Concentration of arsenic species in 42 samples of plant-based foods from Italian market.
